# Genomic interventions for sustainable agriculture

**DOI:** 10.1111/pbi.13472

**Published:** 2020-09-22

**Authors:** Abhishek Bohra, Uday Chand Jha, Ian D. Godwin, Rajeev Kumar Varshney

**Affiliations:** ^1^ ICAR‐Indian Institute of Pulses Research (IIPR) Kanpur India; ^2^ Centre for Crop Science Queensland Alliance for Agriculture and Food Innovation (QAAFI) The University of Queensland Brisbane Qld Australia; ^3^ International Crops Research Institute for the Semi‐Arid Tropics (ICRISAT) Hyderabad India; ^4^ The UWA Institute of Agriculture The University of Western Australia Perth Australia

**Keywords:** genetic gains, genome sequencing, genomic selection, gene editing, speed breeding, varietal turnover, seed replacement

## Abstract

Agricultural production faces a Herculean challenge to feed the increasing global population. Food production systems need to deliver more with finite land and water resources while exerting the least negative influence on the ecosystem. The unpredictability of climate change and consequent changes in pests/pathogens dynamics aggravate the enormity of the challenge. Crop improvement has made significant contributions towards food security, and breeding climate‐smart cultivars are considered the most sustainable way to accelerate food production. However, a fundamental change is needed in the conventional breeding framework in order to respond adequately to the growing food demands. Progress in genomics has provided new concepts and tools that hold promise to make plant breeding procedures more precise and efficient. For instance, reference genome assemblies in combination with germplasm sequencing delineate breeding targets that could contribute to securing future food supply. In this review, we highlight key breakthroughs in plant genome sequencing and explain how the presence of these genome resources in combination with gene editing techniques has revolutionized the procedures of trait discovery and manipulation. Adoption of new approaches such as speed breeding, genomic selection and haplotype‐based breeding could overcome several limitations of conventional breeding. We advocate that strengthening varietal release and seed distribution systems will play a more determining role in delivering genetic gains at farmer’s field. A holistic approach outlined here would be crucial to deliver steady stream of climate‐smart crop cultivars for sustainable agriculture.

## Introduction

The current food production systems are under immense pressure to double their productivity in order to feed the ever‐increasing global population. The current annual yield gains (≈1%) reported for major crops, that is wheat, rice, maize and soybean remain less than what is projected (≈2.4%) to reach the goal of doubling global production (Ray *et al*., 2013). Climate change further aggravates the challenge that the global food production system is facing, and the global yields of aforementioned commodities are likely to reduce in response to every degree Celsius rise in global mean temperature (Varshney *et al*., 2020). Importantly, this remarkable increase in food production has to be achieved with finite or even depleting land resources and water systems, while meeting the demand for ecosystem preservation (Ronald, 2014). Prevalence of extreme weather conditions is projected to influence pests/pathogens dynamics and compromising the plant defence response (Atlin *et al*., 2017).

Traditional plant breeding systems have been in place for decades and delivered a series of widely adopted high‐yielding crop cultivars worldwide. However, longer time invested in variety development and breeding cycles presents a stumbling block to an accelerated response of plant breeders to growing demands for food production (Lenaerts *et al*., 2019). Improving the rates of crop productivity through breeding seeks transformational changes in our current plant breeding operations and decisions (Santantonio *et al*., 2020). Recent progress in genomics technologies has imparted greater strength to the breeders’ toolbox (Bohra *et al*., 2014a,b, 2020; Bohra and Singh, 2015; Varshney *et al*., 2019a). In this review, we highlight the key milestones in plant genome sequencing and discuss how sequencing data have helped illuminate trait architectures and trait alteration. Genomics technologies, when accommodated within new methods like gene editing, rapid generation turnover, including genomic selection and haplotype‐based breeding are likely to increase the rate of genetic gains in breeding programmes. We also underline the significance of varietal release and seed distribution systems in pursuing our goal of sustainable food production.

## Key breakthroughs in plant genome sequencing

A contiguous and well‐annotated genome sequence is the foundation for downstream analyses such as gene/trait discovery, genome dynamics, phylogenetic and evolutionary studies, and better cataloguing of repeat elements (van de Peer, 2018). Advances in DNA sequencing technologies have paved way for decoding of whole genomes for a variety of plant species. Currently, over 400 genomes of different species of land plants are deposited in GenBank (https://www.ncbi.nlm.nih.gov/genome/browse#!/eukaryotes/land%20plants).

In 2000, Arabidopsis became the first multicellular organism sequenced by a multinational consortium using a bacterial artificial chromosome (BAC)‐by‐BAC approach that relies on construction of a minimum tiling path (MTP) based on overlapping BAC clones (AGI, 2000). As reviewed by Kersey (2019), the Arabidopsis genome assembly is of the highest accuracy ‘gold standard’, with the latest version having only 161 gaps. A similar BAC‐based approach was used to sequence the rice crop in 2005 (IRGSP, 2005). A technological breakthrough in genome sequencing was achieved with the whole genome shotgun (WGS) strategy in which the genomic DNA is sheared followed by sequencing and assembly of these fragments. For instance, Tuscan *et al*. (2006) assembled 434.29 Mb genome of poplar (*Populas tricocarpa*) using WGS strategy. However, this strategy yielded a fragmented assembly and proved costly at that time due to its reliance on Sanger chemistry (Bolger *et al*., 2014).

Post‐Sanger sequencing approaches based on next‐generation sequencing (NGS) leveraged the WGS strategy by dramatically improving sequencing throughput at a much reduced time and cost for genome sequencing projects (Varshney *et al*., 2009). The first plant genomes that were created using a combination of Sanger and NGS approaches were grape (Velasco *et al*., 2007) and cucumber (Huang *et al*., 2009a), with short reads generated, respectively, by 454 and Illumina platforms. The first *de novo* whole genome assembly created solely with short‐read technologies was strawberry (*Fragaria vesca*), and the authors used 454, Illumina and SOLiD platforms to decode the whole genome (Shulaev *et al*., 2011).

The NGS platforms have been employed to build reference genome sequences not only for model plants but also for a range of orphan crops such as chickpea (Varshney *et al*., 2013), pigeonpea (Varshney *et al*., 2012) and *Vigna* crops (Kang *et al*., 2014, 2015; Yang *et al*., 2015). Sequencing of reference genomes in different plant species has enabled access to massive genome‐wide genetic markers that are indispensable tool for genomics‐assisted breeding. For example, the reference genome sequences have facilitated development of high‐density genotyping arrays tiled with 1K to 820K single nucleotide polymorphisms (SNPs) spread over the entire genome in important crop plants including rice, wheat, maize, barley, soybean, sorghum, groundnut, chickpea and pigeonpea. (Rasheed *et al*., 2017). Also, mapping‐by‐sequencing approaches guided by the reference genome sequence greatly augment the gene discovery in plants. These mapping‐by‐sequencing approaches have been thoroughly reviewed elsewhere (Davey *et al*., 2011; Schneeberger, 2014).

The Illumina platforms based on sequencing‐by‐synthesis still remain the most preferred NGS system for sequencing. However, the short reads generated by the NGS platforms pose challenges in *de novo* genome assembly, particularly in case of complex genomes with polyploidy, heterozygosity and abundant repeat sequences (Bolger *et al*., 2014; Hu *et al*., 2018). This is evident from the fact that several of the draft genome assemblies built on NGS reads still remain incomplete and fragmented (Paajanen *et al*., 2019). In this context, Belser *et al*. (2018) discussed the varying levels of contiguity in current genome assemblies and they observed that only six plant species have genome assemblies with contig N50 greater than 5 Mb. A constant quest towards overcoming these issues has led to the development of third‐generation sequencing (TGS) technologies (van Dijk *et al*., 2018). The most widely used TGS technologies the PacBio single molecule real‐time (SMRT) sequencing and Oxford Nanopore (MinION/ PromethION) generate read length up to 100 kb and 1 Mb, respectively, with an average of 10–15 kb as against the usual average Illumina read length of 125–300 bp (Hu *et al*., 2018).

The long‐read sequencers in combination with optical maps (Schwartz *et al*., 1993) are being used to generate high‐quality chromosome level genome assemblies (Jiao *et al*., 2017; Paajanen *et al*., 2019; Tang *et al*., 2015). Recently, PacBio RS II system was applied for construction of 2.5 Gb genome assembly of peanut (*Arachis hypogaea*, an allotetraploid) with a contig N50 of 1.5 Mb (Zhuang *et al*., 2019). The long‐range scaffolding techniques such as high‐throughput chromosome conformation capture (Hi‐C) facilitate chromosome‐scale assembly of the contigs. In this respect, recently built genome assemblies of *Brassica rapa* (529 Mb), *B. oleracea* (630 Mb) and *Musa schizocarpa* (587 Mb) showed up to 450‐fold improvement in contiguity over the existing assemblies (Belser *et al*., 2018). Similarly, relative to a new maize genome assembly (PH 207) based on Illumina short read, improved genome sequence of the maize inbred line B73 generated using PacBio RS II system with contig N50 of 1.2 Mb offers a 240‐fold improvement in contig length (Jiao *et al*., 2017). The remarkable improvement in contiguity was achieved in a more recent 2.16‐Gb genome assembly of small‐kernel (SK) maize line based on the long‐read PacBio system, which has a contig N50 of 15.78 Mb (Yang *et al*., 2019). The assembly has 238 gaps as compared to 2,522 of improved B73 assembly. Belser *et al*. (2018) discuss that a combination of Oxford Nanopore, Bionano Genomics, and Illumina could generate a sequence of 500–600 Mb for around US$ 6,000. The cost involved here is remarkably smaller than the 120 Mb genome assembly of Arabidopsis, which was generated at an approximate cost of $100 million over a period of 10 years (Goff *et al*., 2014).

Stimulated by the technological innovations, researchers are undertaking ambitious projects that intend to offer deeper insights into the genomic architectures and evolution (Liu et al., 2019). For example, the 3,000 Rice Genomes Project (Wang *et al*., 2018), 1000 plants project (1 KP, Matasci *et al*., 2014; https://sites.google.com/a/ualberta.ca/onekp/), 3000 chickpea genome sequencing initiative (unpublished) etc. Notable in this context is recently proposed 10 000 plant genomes sequencing project (10 KP) with the aim to deliver more than 10 000 genome sequences across plants and eukaryotic microbes (https://db.cngb.org/10kp/). 10KP is a key component of EarthBioGenome project (https://www.earthbiogenome.org/) with the aim to generate sequence data for 1.5 million known eukaryotic species over a 10‐year period.

## Sequencing multiple genomes to leverage pangenomics

Genetic diversity acts as raw material for crop improvement programmes. According to Mascher *et al*. (2019), exploitation of genetic variation from landraces in crop breeding programmes has met with modest success, with dwarfing genes in rice and wheat and *mlo* alleles in barley being the notable cases. The narrow genetic variation of current crop breeding programmes is because of domestication and modern breeding. In recent years, genome‐scale investigations of wide germplasm panels have served as a great resource to study genomic variation dynamics during domestication and selective breeding (Zhou *et al*., 2015). For instance, recent sequencing of multiple accessions in various crop species in concert with genome‐wide association study (GWAS) has facilitated identification of key genomic regions associated with crop domestication and selection/improvement (Varshney *et al*., 2017).

Availability of the reference genome sequence has stimulated sequencing of multiple accessions of a plant species to enable genome‐scale investigations. For instance, Morrell *et al*. (2012) highlight the importance of comparative genome analyses with the proposition that ‘the future of crop improvement will be centred on comparisons of individual plant genomes’. Sequencing of multiple genomes opens new avenues for pan‐genomic studies that aim to identify core and indispensible genes in crop species. Also, pangenomics has great potential in identifying larger structural variations (SVs) particularly copy number variation (CNV) and presence/absence variation (PAV) that significantly contribute towards phenotypic diversity. Identification of such SVs otherwise remains difficult through analysis of a single reference genome or reference‐based resequencing studies (Tao *et al*., 2019). Sequencing of 292 pigeonpea accessions highlighted the role of evolutionary transitions in shaping structural variation and the association of SVs with the genome regions affected by domestication and modern breeding (Varshney *et al*., 2017). Concerning the identification of the large SVs at chromosomal scale, modern systems based on optical mapping technology such as the Bionano Genomics Saphyr system have remarkable sensitivity towards detection of genome‐wide SVs (https://bionanogenomics.com/support‐page/saphyr‐system/).

More recently, we have proposed a concept of super‐pangenome to capture a complete view of genetic diversity present in a genus. In this approach, first different species‐level pangenomes are constructed and then these pangenomes are combined to obtain a ‘pangenome of pangenomes’ or a genus‐level pangenome. For developing a species‐level pangenome, the most diverse accessions of a species are identified and selected. Then, the genome of one of these accessions is sequenced and assembled *de novo*, which serves as a reference for the mapping of resequencing data from the remaining accessions. The super‐pangenome thus constructed offers better insights into the indispensable genome set and hence has a greater utility for crop improvement **(**Khan *et al*., 2020).

Genomic technologies facilitate efficient characterization and utilization of germplasm stored in global repositories. Creation of subsets of germplasm collections such as core and mini core has been proposed to bring the number of germplasm accessions to manageable level (10% and 1% of the total accessions in core and mini core, respectively) while encompassing high diversity of a species (Upadhyaya and Ortiz, 2001). In the context, DNA marker data were also used for the development of mini core collections in different crops including rice, maize, soybean, peanut, chickpea and pigeonpea (see Guo *et al*., 2014). Cost‐effectiveness of recent high‐throughput genotyping technologies has inspired researchers to perform genome‐wide characterization of global germplasm collections instead of relying on limited subset of collections such as core or mini core. Large‐scale characterization of germplasm collections was carried out in a variety of crops including soybean (14 430 accessions typed with 52 041 SNPs; Bandillo *et al*., 2015) and maize (2815 accessions typed with 681 257 SNPs; Romay *et al*., 2013). A more recent study based on genotyping‐by‐sequencing (GBS) of 22 626 barley accessions from ex situ genebank presents opportunities not only for the discovery of novel beneficial genes but also to take informed decisions for germplasm management (Milner *et al*., 2019). In this context, Mascher *et al*. (2019) recommend to transform genebanks into ‘biodigital resource centres’ which would be instrumental in linking genomic information with the plant performance of each stored accession. Creation of biodigital resource centres will greatly help researchers to make informed choices for pre‐breeding programmes that lead to product delivery. Furthermore, for crop improvement applications, we propose to develop crop diversity panels (CDPs) based on germplasm sequencing data. These CDPs can be evaluated and used for mining the haplotypes for the genes for different target traits. Germplasm lines carrying superior haplotypes can be used in breeding programmes for transferring these unexplored haplotypes and broadening genetic base of elite gene pool.

## Trait discovery in the post‐NGS era

### High‐throughput methods for rapid gene/QTL discovery

Conventional quantitative trait loci (QTL) mapping methods suffer from limited genetic resolution besides having low throughput, being labour‐intensive and time‐consuming in nature. Presence of whole genome sequence in concert with advances in DNA sequencing technologies and computational biology has greatly empowered trait analysis and gene discovery in plants (Jaganathan *et al*., 2020). Last decade has seen emergence of a series of such trait mapping approaches such as SHOREmap, SNP ratio mapping (SRM), next‐generation mapping (NGM), MutMap and QTL‐seq that harness the immense potential of reference genome sequences (Bohra, 2013; Varshney *et al*., 2014; Zhang *et al*., 2019). As a result, candidate QTL regions can be resolved now to a level of few kbs through either sequencing genomes of all individuals of the mapping populations or integrating bulked segregants analysis (BSA) with whole genome resequencing (WGRS). For example, Huang *et al*. (2016) sequenced genomes of more than 10 000 F_2_ individuals from 17 representative hybrid rice crosses and mapped QTLs mostly within 300 kb. The study provided important insights into genomic architecture of heterosis such as occurrence of partial dominance and overdominance at the loci contributing to heterotic advantage. Similar examples include mapping of plant height QTL and *GW5* gene to a 100‐kb (Huang *et al*., 2009b) and 200‐kb region (Xie *et al*., 2010), respectively, in rice and QTL controlling resistance against southern root‐knot nematode within a bin size of 29.7 kb region in soybean (Xu *et al*., 2013) following resequencing of 150, 238 and 246 RILs, respectively. In a biparental population, the mapping resolution of the QTL region achieved by the WGRS was 16.7–144.5 times higher as compared to the conventional QTL mapping using SNP and SSR markers (Xu *et al*., 2013). These studies have resolved candidate genomic regions to a level that is comparable to sequence‐based GWAS of diverse genotypes. For example, GWAS of 302 sequenced genotypes in soybean could narrow down a known QTL region (12‐Mb) for pod dehiscence to a 190‐kb region harbouring 14 genes (Zhou *et al*., 2015).

Alternative approaches based on sequencing of selected/bulked individuals such as QTL‐seq have been widely applied for trait mapping across different crop species owing to its inherent ability to address both qualitative and quantitative traits (Table 1). To this end, Zhang *et al*. (2019) have proposed a new strategy called as quantitative trait gene sequencing (QTG‐seq) to improve genetic resolution achieved by the QTL‐seq. In the QTG‐seq, target QTL selection in the first generation of backcross (BC_1_F_1_) is accompanied by sequencing of selected BC_1_F_2_ pools at relatively high coverage. This allows a quantitative trait to be analysed in a ‘near qualitative’ fashion. Using this strategy, these researchers located a plant height QTL of maize (qPH7) to a 150‐kb genomic interval harbouring a causal gene that codes for an NF‐YC transcription factor.

**Table 1:** A list of some key NGS‐based trait discovery studies in some crops

Crop	Population	Trait analysed	QTL/Gene mapped	References
Rice	NIL‐13B4 × GH998 (F2)	Nitrogen use efficiency	266.5‐kb qNUE6 (LOC_Os06g15370 and LOC_Os06g15420)	Yang *et al*. (2017)
Soybean	Zhonghuang × Jiyu 102(F2)	Seed cotyledon colour	qCC1 (30.7‐kb) and qCC2 (67.7‐kb)	Song *et al*. (2017)
	Jikedou 2 × Huachun 18 (F2)	Phytophthora resistance	146‐kb RpsHC18 (RpsHC18‐NBL1 and RpsHC18‐NBL2)	Zhong *et al*. (2018)
*Brassica napus*	Huyou19 × Purler(F2)	Branch angle	branch angle 1 (BnaA0639380D, a homolog of AtYUCCA6)	Wang *et al*. (2016)
Peanut	ZH8 × ZH9 (RIL)	Testa colour	AhTc1, encoding a MYB transcript factor	Zhao *et al*. (2019)
	TAG 24 × GBPD 4 (RIL)	Rust and late leaf spot resistance	qRust80D_06, qRust90D_06, qRust 80D_07, qRust 90D_07, qRust 80D_08, qRust 90D_08, qRust 80D_09, qRust 90D_09, qLLS70D_08, qLLS 90D_08, qLLS 90D_09	Pandey *et al*. (2017)
	ICGV 00350 × ICGV 97045 (RIL)	Fresh seed dormancy	RING‐H2 finger protein and zeaxanthin epoxidase	Kumar *et al*. (2019)
	Yuanza 9102 × Xuzhou 68‐4 (RIL)	Shelling percentage	10 SNPs in nine candidate genes	Luo *et al*. (2019)
Chickpea	ICC 4958 × ICC 1882 (RIL)	100‐seed weight	*Ca_0436* and *Ca_04607*	Singh *et al*. (2016a)
	ICCV 96029 × CDC Frontier (RIL) ICCV 96029 × Amit (RIL)	Ascochyta blight	Six candidate genes	Deokar *et al*. (2019)
Pigeonpea	ICPL 20096 × ICPL 332 (RIL)	Fusarium wilt and sterility mosaic disease resistance	*C. cajan_03203* and *C. cajan_01839*	Singh *et al*. (2016b)

### Harnessing high‐power mapping resources

**Figure 1:**
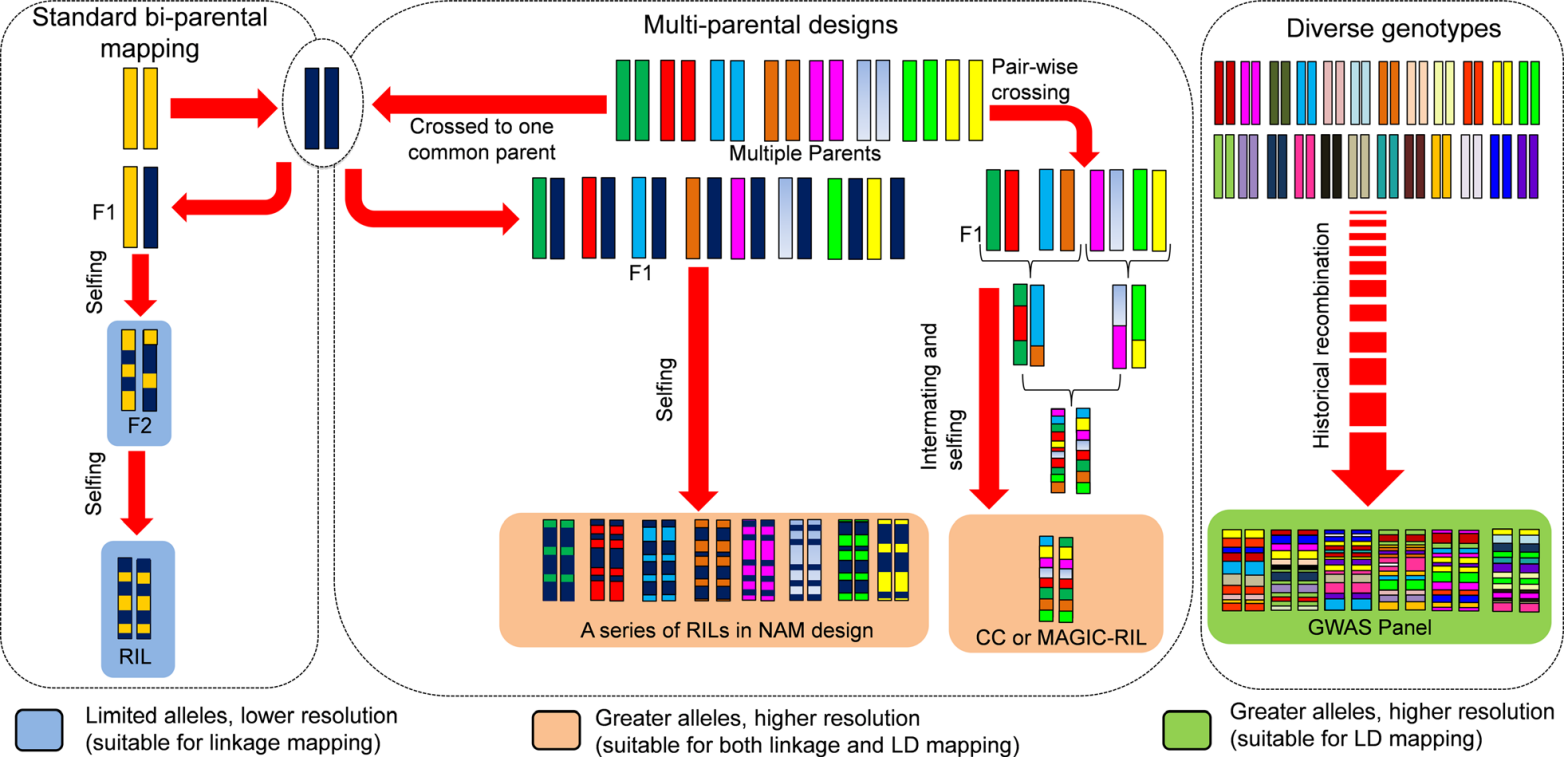
Adoption of new‐generation genetic resources for enhanced trait discovery. The power and precision of trait discovery have improved several folds with increasing adoption of multi‐parent populations and association panel. Importantly, mapping populations derived from multiple founders retain benefits of both linkage analysis and association mapping. CC: Collaborative cross; GWAS: genome‐wide association study; LD: linkage disequilibrium; MAGIC: multi‐parent advanced generation intercross; NAM: nested association mapping; RIL: recombinant inbred line.

With high‐throughput genotyping systems coming within grasp of even small‐scale laboratories, the type of the genetic material being employed for trait mapping studies assumes greater significance (Stadlmeier *et al*., 2018). Biparental QTL mapping has seen tremendous success in understanding the genetic architecture of various important traits in different crop species (Bohra *et al*., 2014b, 2014a). Subsequently, association genetics of diverse panels was proposed to overcome the inherent caveats of biparental analysis such as low mapping resolution, limited allelic diversity and need of artificially created populations. As illustrated in Fig. 1, a greater need to resolve the complex genetic architecture of traits has caused a methodological shift towards broad‐based mapping resources that accommodate diverse founders and abundant recombination events while retaining benefits of linkage‐based designs (Chen *et al*., 2019a). These designs involving multi‐parents impart rich allelic content, higher genetic resolution, large phenotypic diversity and better estimation of allelic effects (Scott *et al*., 2020). Two such designs, that is nested association mapping (NAM) and multi‐parent advanced generation intercross (MAGIC), have been adopted in various crops for trait mapping (Table 2). Even a simplified MAGIC panel with modest population size (394 RILs) is shown to capture nearly 70% of the diversity of German wheat breeding gene pool (Stadlmeier *et al*., 2018). Similarly, sorghum NAM design with 2214 RILs had captured ∼70% of global diversity and shown three times more power than the association panel of the same size to detect QTL for adaptive traits (Bouchet *et al*., 2017).

**Table 2:** Details of multi‐parent populations and their applications in trait mapping in some crops

Crop	Founders involved	Size	Markers assayed	Trait mapped	Approach used	References
*Magic*						
Cowpea	8	305	51, 128 SNPs	Flowering time, growth habit, seed size, maturity, photoperiod	Interval QTL mapping	Huynh *et al*. (2018)
Faba bean (Go¨ttingen Winter Bean Population, GWBP)	11	400	156 SNPs	Morphological traits, fatty acid composition, shoot water content	Association mapping	Sallam and Martsc (2015)
	4	–	875 SNPs	–	–	Khazaei *et al*. (2018)
*Rice*						
Japan‐MAGIC (JAM)	8	981	16 345 SNPs	Days to heading, culm length	GWAS	Ogawa *et al*. (2018)
MAGIC, MAGIC plus, japonica MAGIC, Global MAGIC	8, 16	500–1328	17 387 SNPs	Submergence tolerance, bacterial blight, grain quality	GWAS	
Sorghum	19	1000	79 728 SNPs	Plant height	GWAS	Ongom and Ejeta (2018)
Wheat	8	394	17 267 SNPs	Powdery mildew	Interval QTL mapping	Stadlmeier *et al*. (2018)
	8	1091	90 000 SNPs	Awning		Mackay *et al*. (2014)
	4, 8	1579	1670 DArTs	Plant height and hectolitre weight	Interval QTL mapping	Huang *et al*. (2012)
Maize	8	1636	54 234 SNPs	Days to pollen shed, plant height, ear height and grain yield	Linkage mapping and association mapping	Dell’Acqua *et al*. (2015)
*NAM* ^*^						
Maize (B73)	26	5000	3641 SNPs	Flowering time	Joint linkage analysis and GWAS	Buckler *et al*. (2009)
			1106 SNPs	Northern leaf blight	Joint linkage analysis and GWAS	Poland *et al*. (2011)
			1106 SNPs	Kernel Composition	Joint linkage analysis and GWAS	Cook *et al*. (2012)
			–	Leaf architecture traits	GWAS	Tian *et al*. (2011)
TeoNAM (W22)	6	1257	51 544	Domestication and agronomic traits	Joint linkage analysis and GWAS	Chen *et al*. (2019a)
Soybean (IA3023)	41	5600	5303 SNPs	Grain yield stability	GWAS	Xavier *et al*. (2018)
Wheat (Berkut)	29	2100	800 000 SNPs	–	–	Jordan *et al*. (2018)
Wheat (Asassa)	51	6280	13 000 SNPs	Phenology traits and plant height	GWAS	Kidane *et al*. (2019)
Rice (IR64)	11	1879	7152 SNPs	Days to heading	Joint linkage analysis	Fragoso *et al*. (2017)
Sorghum (RTx430)	11	2214	90 000 SNPs	Flowering time and plant height	Joint linkage analysis	Bouchet *et al*. (2017)
Barley (Barke)	26	1420	27 000 SNPs	Glossy spike, glossy sheath and black hull colour	GWAS	Nice *et al*. (2016)
			5398 SNPs	Yield‐related traits	GWAS	Sharma *et al*. (2018)
Pea (Cameor)	8	927	13 204 SNPs	Seed yield components, seed composition, plant phenology and plant morphology	–	Tayeh *et al*. (2015)
Groundnut (NAM‐Tifrunner & NAM‐Florida‐07)	5, 5	581 496	58 000 SNPs	100‐pod weight and 100‐seed weight	Linkage mapping and association mapping	Gangurde *et al*. (2019)

^*^ Common parent of the NAM population is shown in parentheses.

Nested association mapping comprises a series of connected half‐sib families derived from crossing diverse parents with a common reference parent. MAGIC encompasses cycles of structured intermating among founders and advancement, yielding mosaics of genome blocks from all founders (Huynh *et al*., 2018). The highly recombined nature of these populations has been strongly supported from linkage disequilibrium (LD) patterns inferred from high‐density genotyping (Mackay *et al*., 2014; Ongom and Ejeta, 2018; Scott *et al*., 2020). Inherent to the nature of the mating scheme, recovery of novel QTL combinations is limited in NAM because of the biparental derivation of the constituent RILs. Huang *et al*. (2015) proposed to combine MAGIC with recurrent selection where marker–trait associations (MTAs) are identified and then deployed in the same MAGIC panel to select lines with greater number of positive lines only to be recombined for 2–3 cycles, leading to the development of lines carrying maximum number of positive alleles.

### High‐resolution genome‐wide association studies

Genome‐wide markers such as SNPs/CNVs unleashed from WGRS efforts have greatly empowered GWAS for delineating the smallest possible genome region associated with phenotypic variation in large germplasm sets. Recent instances of WGRS‐based GWAS are worth mentioning in rice (Huang *et al*., 2010; Yano *et al*., 2016), foxtail millet (Jia et al., 2013), soybean (Zhou *et al*., 2015), sesame (Wei et al., 2015), chickpea (Varshney *et al*., 2019b), pigeonpea (Varshney *et al*., 2017) and cotton (Ma *et al*., 2018) for discovering highly resolved MTAs related to traits of economic importance including plant domestication traits (Table 3).

**Table 3:** Some examples of WGRS‐based GWA studies in select crops

Crop	Trait	MTA/QTL/candidate genes identified	SNP/indels	Number of genotypes	LGs/Chromosomes	References
Rice	Agronomic traits	80 significant MTAs	3.6 million SNPs	517	1–12	Huang *et al*. (2010)
Rice	Amylose content and seed length, pericarp colour	*Os03g0407400, Os06g0133000, Os10g0536400*	2.3 million SNPs	203	6, 7, 10	Wang *et al*. (2016)
Rice	Days to heading date, awn length, panicles per plant, plant height, panicle length, spikelet number per panicle, leaf blade width	*LOC_Os01g62780, LOC_Os11g08410, LOC_Os04g52479, LOC_Os08g37890*	426 337 SNPs, 67 544 indels	176	1, 4, 6, 8, 11	Yano *et al*. (2016)
Rice	Grain shape, length and width	*GWi7.1, GWi7.2, GL3.1, GWi5.1, GWi11.1*	2.9 million SNPs, 3.9 million indels	591	3, 5, 7, 10, 11	Misra *et al*. (2017)
Rice	Alkalinity	Eight QTLs	788 396 SNPs	295	3	Li *et al*. (2019)
Rice	17 mineral elements	72 loci	6.4 million SNPs	529	1‐12	Yang *et al*. (2018)
Rice	Heading date, grain mass, straw biomass, harvest index	115 QTLs	∼2 million SNPs	266	1‐12	Norton *et al*. (2018)
Rice	Pericarp colour, amylose content, protein content, panicle number		2 046 529 SNPs	137	2, 3, 5, 6,7, 9	Kim *et al*. (2016)
Rice	Grain width, grain length	MTAs coincided with *GS3, GW5,* and *qGL7, OsFD*	223 743 SNPs	3,010	1, 3, 5, 6, 7, 9, 11	Wang *et al*. (2018)
	Bacterial blight	*Xa26*	148 999 SNPs			
Rice	Seed coat colour, grain length	*Rc* locus, LONG KERNEL 3 gene	889 903 SNPs	365	7, 11	Fuentes *et al*. (2019)
Soybean	Oil content, plant height, domestication traits, pubescence form, flower colour, cyst nematode tolerance, seed weight	–	9 790 744 SNPs, 876 799 indels	302	–	Zhou *et al*. (2015)
Soybean	Salinity tolerance	401 and 328 MTAs for leaf scorch score and leaf chlorophyll content, respectively	5 million SNPs	106	–	Patil *et al*. (2016)
Soybean	84 traits	245 loci	4 million SNPs	809	1–20	Fang *et al*. (2017)
Soybean	Salinity tolerance	51 significant MTAs	3.7 million SNPs	234	1, 2, 3, 5, 6, 8, 14,15,16, 18, 19, 20	Do *et al*. (2019)
Chickpea	Ascochyta blight	AB4.1 and 12 candidate genes and 20 significant SNPs	144 000 SNPs	132	4	Li *et al*. (2017)
Chickpea	Yield‐related traits under drought stress	38 significant SNPs	144 777 SNPs	132	3, 4, 5, 6	Li *et al*. (2018)
Chickpea	Traits related to drought and heat stress	262 MTAs and several candidate genes including TIC, REF6, aspartic protease, cc‐NBS‐LRR, RGA3, Ca_13671, Ca_13939	3.65 million SNPs	429	–	Varshney *et al*. (2019)
Pigeonpea	Agronomic traits	241 MTAs, homologs of LIGULELESS1, SHATTERING1 and EARLY FLOWERING3 (ELF3)	15.1 million SNPs, 2.1 million indels	292	1–11	Varshney *et al*. (2017)
Common bean	Phenological traits and yield and yield‐related traits, anthracnose resistance	505 MTAs	4.8 million SNPs	683	1–11	Wu *et al*. (2020)
Linseed	Seed size and seed weight	13 candidate genes	674 074 SNPs	200	1, 4, 5, 6, 7, 9, 11, 12, 14, 15	Guo *et al*. (2020)
*Brassica napus*	Seed yield, silique length, oil content and seed quality	60 loci	670 028 SNPs	588	A08, A02, A09, C02, C03, C07	Lu *et al*. (2019)
*Brassica napus*	Flowering time	*FLOWERING LOCUS T, FLOWERING LOCUS C*	2 753 575 SNPs	991	A02	Wu *et al*. (2019)

A GWA study of more than 500 sequenced landraces in rice elucidated a total 80 MTAs for 14 different traits related to grain yield and quality, physiology and drought stress (Huang *et al*., 2010). Similarly, GWAS based on WGRS of 176 rice accessions uncovered four genes viz., *LOC_Os01g62780 (*days to heading date), *LOC_Os11g08410 (*plant height and panicle length)*, LOC_Os04g52479 (*panicle no. per plant and spikelet number per panicle) and *LOC_Os08g37890* (awn length) (Yano *et al*., 2016). In cotton, GWAS of a core collection of cotton with 419 lines allowed fine‐dissection of fibre‐related traits and the flowering time trait (Ma *et al*., 2018).

In legumes, WGRS‐based GWAS has been successfully applied for delineating new QTLs/candidate gene(s)/MTAs along with validating the loci identified previously through QTL mapping or association studies. In soybean, GWAS on 302 sequenced genotypes identified several new MTAs that remain congruent with the previously identified QTLs controlling a range of domestication‐related traits (Zhou *et al*., 2015). Another GWA study in soybean with WGRS‐SNPs on 106 lines revealed 401 and 328 SNPs significantly associated leaf scorch score (LSS) and leaf chlorophyll content, respectively, contributing to salinity tolerance (Patil *et al*., 2016). Interestingly, the most significant SNP related to LSS was pinpointed in *GmCHX1* gene, which explained 63% variation of the phenotype (Patil *et al*., 2016). Likewise, GWAS of 234 lines elucidated genomic architecture of salinity tolerance in soybean with significant MTAs for leaf scorch score, chlorophyll content ratio, leaf sodium content and leaf chloride content (Do *et al*., 2019).

A recent GWA study of a 429‐line global reference set of chickpea elucidated important candidate genes underlying 262 MTAs controlling various traits that confer heat and drought stress tolerance (Varshney *et al*., 2019b). In legumes, other high‐resolution trait mapping studies combining GWAS and WGRS were performed for drought stress in 132 lines of chickpea (Li *et al*., 2018), yield/seed traits and anthracnose resistance in 683 lines of common bean (Wu *et al*., 2020) and adaptive traits in 292 pigeonpea accessions (Varshney *et al*., 2017). The GWAS has been greatly benefited by the enhanced marker density of WGRS, and however, the mapping resolution of GWAS depends on the extent of LD and recombination rate, which vary in different plant species (self‐pollinated or cross‐pollinated), and among different populations (wild, landraces and improved cultivars) and within the genome (euchromatin and heterochromatin regions) of a given species (Chang *et al*., 2018; Zhou *et al*., 2015).

### Genomics‐informed gene editing

Gene editing technologies include a number of powerful tools to directly change genetic sequences in coding and/or regulatory regions to create new alleles, most effectively without introducing new transgenes (Zhang *et al*., 2018). The most frequently applied techniques include CRISPR (Clustered Regularly Interspaced Palindromic Repeats) or TALEN (Transcription Activator Like Effector Nucleases), with the CRISPR/Cas system being the simplest gene editing system to apply. The basic techniques and applications for crop gene editing have been well‐described elsewhere (Chen *et al*., 2019b; Schindele *et al*., 2020; Zhang *et al*., 2018; Zhang *et al*., 2019). As a complementary tool to genomics, gene editing can resolve questions as to gene identity and function, as well as provide novel allelic variants not available within the crop species or interfertile relatives in the domesticated primary or wild secondary gene pools.

Gene editing techniques can be used to knockout genes, usually by inducing small insertions or deletions, which lead to frameshift mutations causing premature stop codons. The most frequent approach relies on non‐homologous end joining (NHEJ) edits. This type of editing targets a region of the coding regions or sometimes a regulatory sequence. CRISPR/Cas9 induces a double‐strand break in the DNA and relies on the cell’s endogenous DNA repair mechanisms to religate the broken strands. While majority of DNA repair mechanisms are intrinsically accurate, it is error prone, and it is these errors that make new alleles. As already stated, these frequently result in new non‐functional alleles. However, at a low frequency they may simply cause a single non‐synonymous amino acid change. These repair mechanisms can also delete nucleotide in multiple of three, which will lead to deletions of amino acids or even peptides and these may alter the gene expression without actually creating a null allele. In a number of jurisdictions, including the United States, Australia, Japan and Argentina, these types of changes give rise to plants that are not regarded as transgenic and hence can be rapidly used in breeding programmes.

It is also possible to apply gene editing such that a specific repair template is used to change the coding or regulatory sequence in a specific manner. These homology directed repair (HDR) edits can be extremely powerful tools to edit genes and create novel variants. New or different amino acids may be introduced, from a single amino acid up to hundreds of amino acids, depending on the templates used. However, there are considerable restrictions to their usage, and in many jurisdictions, the introduction of any new DNA to the host is sufficient for these to be classified as transgenic. In others, such as in the United States and Japan, they may be considered as non‐transgenic on a case‐by‐case basis.

Biallelic editing using CRISPR/Cas9 and Mendelian inheritance of these edits was first reported in *Arabidopsis* and crop plants including rice (Zhang *et al*., 2014) and tomato (Brooks *et al*., 2014). This paved the way for gene editing to be broadly applied across species. It was soon demonstrated that multiple genes and gene combinations could be edited simultaneously. Wang *et al*. (2014) demonstrated that simultaneous editing of 3 homeoalleles in hexaploid wheat could be performed to develop powdery mildew resistance in wheat. Indeed, the wild relatives of crops can also be edited to increase their utility as either new crops or sources of novel genetic variation. *Solanum pimpinellifolium*, a wild relative of cultivated tomato, was edited at six independent loci to produce plants more closely resembling the domesticated *S. lycopersici* for key fruit traits. These gene‐edited plants produced more flowers and fruits, with larger fruits, fewer seeds and higher lycopene content in the fruits than the wild species (Zsögön *et al*., 2018). It quickly became evident that producing gene‐edited plants became more straightforward than detecting edited plants, particularly when the altered phenotype was not evident visually. Various groups have developed rapid phenotyping tests to more efficiently screen plants for the most desirable edit(s) (Peng *et al*., 2018).

Used in concert with genomics techniques, gene editing is a particularly elegant tool for gene discovery. Indeed, many gene‐edited crop plants have been produced based on either gene identification in other species, quite often in model species. Where genomic approaches have been used for gene discovery purposes, it can be a laborious process to increase the recombination events around the desired haplotype. It is not infrequent that a region associated with a trait or QTL may be in the order of 100‐500 genes, dependent on the LD in a species/population. Hence, the ability to identify the true causative gene among many potential candidates can be time‐consuming. The use of classical transgenics has been useful and informative, yet imprecise because of variables such as position effect and gene dosage where the transgene inserts into the host genome.

The availability of gene editing techniques offers considerable advantages in identifying candidate genes and genetic interactions to elucidate gene action in the understanding of QTL regions. The edit(s) can be made in the actual gene, and hence, there are no position or dosage effects. Gene expression can be totally knocked out, which has previously been difficult using RNAi approaches, which usually lead to a diminution of gene expression but rarely to zero (Eamens *et al*., 2008). This also means that editing of candidate genes enables clear identification of single gene action. As another advantage, multiple candidate genes can be targeted in a single experiment. For example, three genes, A, B and C, can be edited and the independent progenies will include lines with the individual genes edited and all possible combinations (A + B, B + C, A + C, A + B + C) provided sufficient lines are produced. This can be extremely effective to identify candidate genes in a linkage block, to elucidate specific interactions in a multigene pathway, to uncover evidence of epistasis and to determine instances of pleiotropy and close linkage.

A current limitation of the power of gene editing is the reliance on tissue culture techniques for editing to be performed in most crop species. As a result, gene editing can be extremely genotype limited. The development of tools and broadly applicable means to edit genes without the need of *in vitro* plant regeneration will enable the application of gene editing significantly more efficiently and rapidly. A number of techniques are currently being used to overcome the genotypic bottleneck of gene editing, as reviewed in Hickey *et al*. (2019).

## Breeding strategies to deliver higher genetic gains

Genetic gains from a selection programme can be expressed in the form of breeder’s equation, that is Δ*G* = *R* = *h* ^2^ *S* = *σa* × *i* × *r/L*. Following the equation, the gain (Δ*G*) or response to selection (*R*) can be improved by tweaking additive genetic variation (*σa*) or narrow sense heritability (*h* ^2^), selection intensity (*i*) and selection accuracy (*r*) and length of the breeding cycle (*L*). In the following section, we discuss the new breeding methodologies that address different components of the breeder’s equation and improve the rate of genetic gain in a breeding programme.

### Genomic selection

The paradigm ‘genotype once phenotype many times’ has dominated genetic studies for the past two decades owing to the high cost of genotyping. The increased availability of homozygous immortal genetic populations allowing replications across time and locations further fuelled this paradigm (Srivastava *et al*., 2017). With the development of NGS, genome‐wide marker assays are now affordable, accurate and high throughput. However, acquisition of accurate and precise phenotyping data on sizeable individuals presents a major bottleneck in plant breeding programmes. This has stimulated adoption of new breeding techniques that optimize phenotyping requirements for improving complex traits controlled by a number of small‐effect QTL (Akdemir and Isidro‐Sanchez, 2019).

Genomic selection (GS) improves genetic gain by enhancing selection intensity (i) and selection accuracy (*r*) and reducing the breeding cycle length (*L*). GS predicts genetic merit of unobserved phenotypes from target population based on the breeding values (GEBVs) computed from genome‐wide information of a training set that has been scored phenotypically.

Since the concept was originally proposed by Meuwissen *et al*. (2001), GS implementation has seen tremendous success in animal breeding, and some of the GS studies show 50%–100% increase in genetic gain per year for yield traits in dairy cattle and 35% increase in pig breeding programme (Edwards *et al*., 2019). The key factors underlying success of GS in animal breeding are greater economic returns from early selections and reduced generation intervals, weaker genotype–environment interactions (G × E) and easily controllable environments, higher individual value, large training populations with stronger genetic relatedness between training and breeding individuals, access to both cost‐efficient genotyping systems and historical phenotypic records, greater significance of additive genetic effects and the straightforward incorporation of existing best linear unbiased predictor (BLUP)‐based approaches into the prediction models (Jonas and de Koning, 2013; Santantonio *et al*., 2020; Xu *et al*., 2020). In plants, recent simulation and empirical evidence has established superiority of GS over traditional selection methods including phenotypic, pedigree and marker‐assisted selections (Crossa *et al*., 2017). For long‐term selection gains in hybrid breeding, genome‐wide predictions have been used for identification of heterotic groups and establishment of heterotic patterns in various crops including wheat (Zhao *et al*., 2015), rice (Beukert *et al*., 2017) and pigeonpea (R. K. Saxena, et al., Unpublished data)

A variety of factors are known to influence GS prediction accuracy, that is the degree to which GEBVs relate to estimated genetic values (Akdemir and Isidro‐Sanchez, 2019), which include training population size, relatedness between training and test individuals, DNA marker type and density, trait architectures and heritability, statistical models and population structure (Roorkiwal *et al*., 2018; Thorwarth *et al*., 2017; Xu *et al*., 2020; Zhang *et al*., 2017). Optimization of these factors has shown improvements in GS prediction accuracies.

Studies suggest that using multi‐environmental settings and incorporating GXE interactions into GS models improve prediction accuracies (Jarquín *et al*., 2014; Roorkiwal *et al*., 2018; Sukumaran *et al*., 2017). Though GS unlike MAS does not need a set of DNA markers associated with the trait, incorporating information about the significant markers is shown to improve prediction accuracies (Spindel *et al*., 2016). In a recent GS study in chickpea, Li *et al*. (2018) obtained twofold improvement in prediction accuracy with a subset of SNPs informed by GWAS as compared to using all WGRS‐SNPs. Of the various models used to predict the genetic worth of unobserved individuals, GBLUP remains the most extensively used (Table 4). Further improvement in prediction accuracy is expected with advances in high‐throughput phenotyping such as hyperspectral imaging (Crossa *et al*., 2017). However, application of deep machine‐learning methods for genome‐wide prediction awaits further research.

**Table 4:** Genome‐wide predictions for various traits in crops

Crop	Training population	Markers used	Traits analysed	Predictive ability/Prediction accuracy	Model	References
Wheat	1100 PYT lines (F_3:6_)	27 000 SNPs	Grain yield	0.17–0.28	GBLUP	Belamkar *et al*. (2018)
	10 375 lines	18 101 SNPs	Grain yield, relative maturity, glaucousness and thousand‐kernel weight	0.59–0.98	Maximal model (GBLUP)	Norman *et al*. (2018)
	330 lines from HarvestPlus Association Mapping (HPAM) panel	24 497 SNPs	Grain zinc and iron concentrations, thousand‐kernel weight and days to maturity	0.324–0.76	GBLUP using the reaction norm model	Velu *et al*. (2016)
	208 lines	6211 DArTseq‐SNPs	Grain yield, thousand‐grain weight, grain number, days to anthesis, days to maturity, plant height and normalized difference vegetation index at vegetative and grain filling	0.34–0.68	GBLUP	Sukumaran *et al*. (2018)
	287 advanced elite lines (WAMI panel)	15 000 SNPs	Grain yield, thousand‐grain weight), grain number, thermal time for flowering	0.27–0.63	GBLUP	Sukumaran *et al*. (2017)
	1378 breeding lines	–	Grain yield and yield stability	up to 0.54	Reaction norm models	Jarquín *et al*. (2017)
	2992 F2:4 lines	25 000 SNPs	Grain yield	0.125–0.127	GBLUP	Edwards *et al*. (2019)
	2325 inbred lines	12 642 SNPs	Fusarium head blight, *Septoria tritici* blotch	up to 0.6	RR‐BLUP, Bayes Cπ, RKHS, EG‐BLUP	Mirdita *et al*. (2015)
Soybean	301 elite breeding lines	52 349 SNPs	Grain yield	0.43–0.68	G‐BLUP, G°G, Kaa, G_G°G, G_Kaa	Jarquín *et al*. (2014)
Maize	169 doubled haploid lines and 190 testcrosses	20 473 SNPs	Grain yield, plant height, anthesis‐silking interval, normalized difference vegetative index (NDVI), the green leaf area duration (GLAD)	0.16–0.48	rrBLUP	Cerrudo *et al*. (2018)
	4120 lines from 22 biparental populations	200 SNPs	Grain yield, anthesis date, plant height	0.18–0.38	rrBLUP	Zhang *et al*. (2017)
	284 inbred lines	55 000 SNPs	Female flowering, male flowering, grain yield, anthesis‐silking interval	0.28–0.84	Bayesian LASSO (BL), radial basis function neural network (RBFNN), reproducing kernel Hilbert space (RKHS)	Crossa *et al*. (2014)
Barley	750 lines	11 203 SNPs	Earing, hectolitre weight, spikes per square metre, thousand‐kernel weight and yield	0.31–0.71	GBLUP	Thorwarth *et al*. (2017)
Pea	315 RILs	400–500 SNPs	Grain yield	0.4–0.5	BL, rrBLUP, support vector regression (SVR)	Annicchiarico *et al*. (2017)
	339 accessions	13 200 SNPs	Thousand seed weight, the number of seeds per plant and the date of flowering	up to 0.83	Kernel partial least squares regression (kPLSR), least absolute shrinkage and selection operator (LASSO), genomic best linear unbiased prediction (GBLUP), BayesA and BayesB using	Tayeh *et al*. (2015)
Chickpea	320 breeding lines	3000 DArTs and DArTSeq‐SNPs	Days to flowering, days to maturity, 100‐seed weight and seed yield	0.138–0.912	RR‐BLUP, Kinship Gauss, BayesCp, BayesB, BayesLASSO, and Random Forest	Roorkiwal *et al*. (2016)
	320 breeding lines	90 000 SNPs	Yield and yield‐related traits	–	Multiplicative reaction norm model (MRNM)	Roorkiwal *et al*. (2018)
	132 advanced breeding lines and varieties	147 777 SNPs	Yield and yield‐related traits	0.25	RR‐BLUP, Bayesian LASSO, and Bayesian ridge regression (BRR)	Li *et al*. (2018)

Since the public breeding programmes in developing countries are severely constrained by the lack of resources and appropriate technical skills, Santantonio *et al*. (2020) recommend a phased GS implementation in order to adopt GS as a routine strategy for crop breeding. The initial phase involves informatics development and genotyping of lines that are the most relevant to breeding programmes such as the lines entering in the variety release system. In the second phase, GS is applied to enhance selection intensity in varietal development programmes, while the final phase focuses on rapid cycle recurrent selection. Such optimized approaches that allow the efficient use of recourses and technical expertise will be crucial for large‐scale implementation of GS in breeding programmes of public sectors.

### Rapid generation turnover

Traditional plant breeding methods have delivered a series of high‐yielding crop cultivars suited to diverse agro‐climatic conditions worldwide. However, reliance of these traditional methods on repeated cycles of crossing and inbreeding requires 10–15 years for developing and releasing a new crop cultivar. The lengthy crop breeding cycles have been described as a ‘high entry barrier’ in accelerating crop research with modern tools and technologies (Watson *et al*., 2018).

As mentioned in the previous section, manipulating parameters of breeder’s equation could improve rate of genetic gain. However, approaches that could shorten the length of breeding cycle are considered to substantially influence Δ*G* in comparison with manipulating other parameters of the equation (Cobb *et al*., 2019; Li *et al*., 2018). The protocols collectively grouped under ‘speed breeding’ (SB) aim to accelerate plant development and shorten breeding cycle time *via* optimizing *in vivo* growth conditions such as light, photoperiod, temperature, humidity in combination with enhanced plant density and early seed harvesting (Ghosh *et al*., 2018). To reduce time to anthesis, application of *in vitro* protocols is recommended for germination of immature seeds (Croser *et al*., 2016). Optimized SB recipes have proven to be effective in different crops including wheat (Watson *et al*., 2018), barley (Hickey *et al*., 2017), chickpea (Samineni *et al*., 2020) and pea (Mobini and Warkentin, 2016) for obtaining multiple generations in a single year. The technology has great potential to accelerate breeding programmes for rapid delivery of crop cultivars. However, the SB protocols do not represent a ‘one size fits all’ system and need to be tailored according to both crop behaviour and resources at hand. Also, further experimentation is needed to extend these protocols to short‐day plants such as rice, maize (Watson *et al*., 2018). In the context, preliminary results in pigeonpea, a short‐day plant, show the possibility to achieve four generations per year using immature seed harvest, single pod descent and controlled light/humidity conditions (Saxena *et al*., 2017, 2019). Exhaustive survey of the photoperiod response of different genotypes sets an essential prerequisite for adoption of SB protocols in crop research and breeding. Also, genotype independence of these protocols still remains to be established, which will in turn confirm the broader applicability of this technique across diverse crops and crop genotypes.

The unique abilities of the GS and SB to shorten breeding cycle time could be harnessed synergistically to further enhance the rate of genetic gain per unit time, a strategy termed as ‘SpeedGS’ (Voss‐Fels *et al*., 2019). Simulation study by Voss‐Fels *et al*. (2019) compared different scenarios [phenotypic selection (PS) and GS alone and SpeedGS] and the authors observed that schemes integrating GS with SB witness 30% more genetic gain after 30 years as compared to the PS alone. However, authors suggested introgression of new diversity into the SpeedGS scheme in order to sustain the gain in long term. A simulation study in fescue also reported higher genetic gains in speedGS than that of PS (Jighly *et al*., 2019). Importantly, the improvement in genetic gain was higher in the case of low‐heritability traits and with higher number of SB cycles. Recent empirical evidence in wheat demonstrates the potential of SpeedGS for rapid population improvement where phenotyping of SB traits in combination with multivariate GS could guide the selection of lines for field trials or next breeding cycle (Watson *et al*., 2019). These recent studies highlight the immense scope for ‘customizing the breeding pipelines’ (Voss‐Fels *et al*., 2019) in order to accommodate SB and GS to achieve higher rate of genetic gains in crop breeding programmes.

### Haplotype‐based breeding

Agricultural traits are controlled by genomic loci that are ‘compound’ in nature. In other words, these loci contain several candidate genes that exert influence of varying degree and nature on the associated phenotype. Because of this, unexpected outcomes are often witnessed while transferring genomic regions through routine MAS/MABC technique. In the context, Bevan *et al*. (2017) have proposed a haplotype‐based approach that capitalizes on the deluge of whole genome sequencing data and extensive phenotypic records in order to allow such ‘compound’ loci incorporated efficiently in breeding programmes. Here, different haplotypes for the given locus may be defined as combinations of genes and genetic polymorphisms that are inherited together.

Presence of multi‐year and multi‐location phenotypic data enables a genome‐scale analysis of haplotypes for their phenotypic validation. As has been demonstrated in rice, a panel of sequenced lines capturing the maximum diversity is deemed suitable for phenotypic validation of haplotypes defining key traits (Abbai *et al*., 2019). A similar *haplo‐pheno* analysis in pigeonpea validated superior haplotypes of three genes for drought tolerance that were identified by mining of the WGRS data set and candidate gene‐based association analysis (Sinha *et al*., 2020). The study also identified a set of promising lines carrying these superior haplotypes. Introgression of superior haplotypes in breeding has been referred as haplotype‐based breeding (Sinha *et al*., 2020; Varshney *et al*., 2020).

Tracking sequence variation that marks the validated haplotype, in breeding programme will facilitate synthesis of an ideal line harbouring novel combinations of such established haplotypes. Retrospectively, targeted analysis of superior haplotypes across mega‐varieties may help revealing combinations of superior haplotypes that explain the genetic basis of the high‐performance of these lines. In pigeonpea, Sinha *et al*. (2020) found complete absence of superior haplotypes for drought tolerance in popular varieties Maruti (ICP 8863) and Jagriti (ICPL 151), thus offering possibilities for further improvement of such high‐yielding varieties. In parallel, increasing sequencing data on wild relatives will aid in the discovery of new haplotypes that the cultivated pool currently lacks.

### Accelerating rates of varietal and seed replacements

Since high‐yielding semi‐dwarf varieties of wheat and rice heralded the ‘Green Revolution’ in the late 1960, mega‐varieties of major staple crops have received widespread adoption (Pingali, 2012; Singh, 2017; Singh *et al*., 2020). Farmers cultivate these old varieties and landraces for decades, particularly in the under‐developed and developing countries in South Asia and sub‐Saharan Africa. The average age of rice varieties in South Asia (14–25 years; Pandey *et al*., 2015) and sub‐Sahara Africa (15.8 years; Walker *et al*., 2015) confirms this trend. A recent study reported cultivation of even 25‐year‐old wheat varieties in major wheat‐growing states in India (Pavithra *et al*., 2017). Breeding techniques have yielded more than 500 maize varieties in sub‐Saharan African regions. Nevertheless, old maize cultivars remain predominant in the farmer’s field across these regions (Abate *et al*., 2017). In case of maize, the average age of cultivars is 14–24 years in Kenya (Walker *et al*., 2015) and 18 years in sub‐Saharan Africa (Witcombe *et al*., 2016).

According to Singh *et al*. (2020), farmer’s preference for older varieties in India is evident from the quantity of breeder seed (BS) indented. It is observed that yield gains of these obsolete cultivars are severely deteriorating due to growing prevalence of extreme weather conditions and resurgence of new diseases and pests (Atlin *et al*., 2017). In such scenario, varietal replace race (VRR) could be a key driver for accelerating the genetic gain (Spielman and Melinda, 2017). The VRR reflects the pace with which new varieties with enhanced yield and resilience are deployed at farmers’ field to replace the existing cultivars.

Farmers in the USA, China and Europe have now higher accessibility to newly released varieties that are better adapted to the current situations (Atlin *et al*., 2017). The varietal turnover period of hybrid maize in the USA has been reduced to 3–4 years from that of eight years in the early 1990s (Abate *et al*., 2017). Likewise, variety turnover time in tropical countries viz., Mexico, Brazil and Argentina is reported to be 3–4 years in comparison with 5–7 years in the subtropics and in Asia (Abate *et al*., 2017). The high average age of the predominant hybrids (13 years) in sub‐Saharan Africa has greatly hampered achieving potential yield gain in maize (Abate *et al*., 2017). A comparative assessment of cultivar adoption among three African countries suggested Ethiopia as having the lowest percentage of farmers (25%) adopting improved maize cultivars, while Tanzania (58%) and Malawi (61%) had the higher proportions (Westengen *et al*., 2019). Replacing older maize varieties with improved drought‐tolerant varieties is reported to enhance maize yields and reduce poverty by 13.3% and 12.9%, respectively, in rural Nigeria (Wossen *et al*., 2017). Higher genetic gains and resistance levels from higher VRR have been evident from the data of semi‐dwarf high‐yielding wheat varieties adopted during 1960 and 1970 in India (Byerlee and Heisey, 1990). Farmers’ awareness about improved varieties showed positive association with the adoption of improved pulses’ varieties in Tanzania and Ethiopia (Abate *et al*., 2012; Amare *et al*., 2012). For replacing the existing popular variety, modern plant breeders have to develop market‐oriented ‘product profiles’ with clearly defined ‘trait package’ that may help encouraging farmers to accept new variety (Cobb *et al*., 2019; Ragot *et al*., 2018; https://excellenceinbreeding.org/blog/product‐profiles‐are‐blueprint‐breeding‐impact#). Engaging farmers in selection in crop breeding trials and nursery through participatory plant breeding and participatory varietal selection could also contribute to enhancing VRR (Atlin *et al*., 2017).

Like VRR, availability of quality seed and seed replacement ratio (SRR) could contribute to improving genetic gain. Low SRR in India despite increased availability of quality seed is due to farmers being accustomed to use >70% farm‐saved seed for raising the succeeding crop (Pattanaik, 2013). Recently, the SRR of various crops including cereals, pulses and oilseeds has seen a notable rise in India following implementation of national seed policy (2002) that encouraged farmer’s access to seeds of newly developed varieties and replacement of old varieties (Singh *et al*., 2017). In this context, recent initiatives by Department of Agriculture Cooperation and Farmers Welfare (DACFW), India and Indian Council of Agricultural Research (ICAR), India on enhancing availability of quality seeds to farmers are noteworthy, such as creation of seed hubs for major pulse, millet and oilseed crops.

Seed certification being an essential step for seed quality control (QC) merits attention of both public and private agencies. Flexible systems for seed certification are warranted such that of quality declared system (QDS) adopted in countries such as Kenya and Zambia, where seed certification is licensed to private institutions (Varshney *et al*., 2018). With the increasing number of cultivars being released in different crops, the morphological descriptors used for discriminating these become increasingly limited and the procedure of testing the genetic purity of cultivars (grow‐out test) is time‐consuming, costly and prone to environmental fluctuations. In this context, modern genomic technologies owing to their high throughput and environmental independent nature facilitate cost‐effective and reliable examination of genetic purity and identity, complementing the quality assurance (QA) and quality control (QC) system of various seed companies and seed certification agencies (Bohar *et al*., 2020). For instance, low‐density SNP assays optimized for several crops facilitate data generation of 10‐100 SNPs in US $ 1–5 per sample including DNA extraction (http://cegsb.icrisat.org/high‐throughput‐genotyping‐project‐htpg/). More recently, specific‐locus amplified fragment sequencing (SLAF‐seq) technology and customized SNP array (maizeSNP3072) were optimized to support varietal identification in soybean (Zhang *et al*., 2020) and maize (Tian *et al*., 2015), respectively. Similarly, Pembleton *et al*. (2016) demonstrated the utility of the GBS technology in testing seed purity of ryegrass cultivars by detecting the mislabeled seed lots. Recognizing the immense potential of genomic technologies to address seed quality‐related issues, the International Union for the Protection of New Varieties of Plants (UPOV) has also set guidelines for using marker technologies in distinctness, uniformity and stability (DUS) testing (https://www.upov.int/edocs/tgpdocs/en/tgp_15.pdf).

Collectively, increased genetic gain for meeting the rising demand of food grain could be achieved through a holistic approach covering re‐orientation of public–private programme related to seed business, implementation of sound seed policies and farm innovation to farmers’ awareness (Alwang *et al*., 2017; Siddique *et al*., 2012). As has been adopted recently in India, seed production of obsolete cultivars should be discouraged through denotifying/decertification the obsolete varieties or varieties older than 10 years (Shiferaw *et al*., 2013). Farmers’ access to newly developed varieties also depends upon the streamlining and accelerating the varietal release and notification processes. Extension activities also need attention for disseminating the information on the latest released varieties with the package of practices clearly highlighting their unique advantages over the obsolete varieties (Atlin *et al*., 2017; Singh *et al*., 2020).

## Conclusion and prospects

Recent progress in genomics research has provided geneticists, biologists and breeders with a number of modern tools and technologies that impart precision and efficiency to breeding programmes. Reference genome assemblies are increasingly becoming available, and consequently, methods of gene discovery and trait manipulation have been transformed. Genomics research is also advancing gene editing methods in plants for elucidating candidate genes and genetic interactions.

Breeding techniques such as marker‐assisted back crossing (MABC) are suited more for defect elimination of mega‐varieties; however, enhancing genetic gains per unit time warrants rapid population improvement informed by genome‐wide predictions and associations (Varshney *et al*., 2019a). Increasing access to the deluge of multi‐omics information and high‐dimensional phenotypic data are also revealing the potential challenges associated with handling and interpretation of the data. Plant breeders need to be trained adequately, and this would play a significant role in embracing more sophisticated approaches such as systems biology‐driven breeding for crop improvement (Lavarenne *et al*., 2018). Adopting these new approaches would fast track the development of climate‐smart cultivars. Notwithstanding this, enhanced variety release and seed distribution systems remain instrumental to deploy these new climate‐smart cultivars at the farmers’ field, concurrent with the replacement of old obsolete cultivars. Such coordinated efforts involving multiple disciplines would be central to provide solutions for sustainable agriculture.

## Conflict of interest

The authors declared that they have no conflict of interest.

## Author contributions

AB and RKV jointly developed the conceptual structure. AB prepared the original draft. UCJ and IDG contributed specific sections. RKV edited manuscript. All authors read and approved the final manuscript.
